# Myopia Control With Multifocal Lens in School-Aged Children: A Meta-Analysis

**DOI:** 10.3389/fped.2022.889243

**Published:** 2022-06-20

**Authors:** Meilan Chen, Lu Xu, Hongyang Li, Fengping Cai, Hao Wang, Chun Hu, Yi Wu

**Affiliations:** ^1^Department of Ophthalmology, Guangdong Second Provincial General Hospital, Guangzhou, China; ^2^Institute for Brain Science and Rehabilitation, South China Normal University, Guangzhou, China

**Keywords:** multifocal lens, bifocal lens, peripheral additional lens, myopia control, meta-analysis, children

## Abstract

**Background:**

Myopia is one of the most common eye diseases in school-aged children. Multifocal lens (MFL) is one of the interventions that has being widely applied to control the progress of myopia. However, the treatment effects of MFLs in school-aged children require to be systematically evaluated.

**Methods:**

A systematic analysis on qualified randomized controlled trials (RCTs) in which MFLs were prescribed as the intervention and single-vision lenses (SVLs) as the control was conducted. The treatment effects referring to the mean differences in spherical equivalent refraction (SER) and axial length (AL) between MFLs and SVLs groups were analyzed.

**Results:**

With annual visit (3-years follow-up), the weighted mean differences (WMDs) in SER between MFLs and SVLs were 0.29 D (95% CI, 0.21 ∼ 0.37, *p* < 0.00001), 0.46 D (95% CI, 0.32 ∼ 0.60, *p* < 0.00001), and 0.64 D (95% CI, 0.40 ∼ 0.88, *p* < 0.00001) at the first, second, and third year; in AL were −0.12 mm (95% CI, −0.14 ∼−0.11, *p* < 0.00001), −0.19 mm (95% CI, −0.22 ∼−0.16, *p* < 0.00001), and −0.26 mm (95% CI, −0.31 ∼−0.21, *p* < 0.00001) at the first, second, and third year. With 6-months interval trials (2-years follow-up), the WMDs in SER from MFLs were 0.14 D (95% CI, 0.08 ∼ 0.20, *p* < 0.0001), 0.19 D (95% CI, 0.11 ∼ 0.28, *p* < 0.0001), 0.24 D (95% CI, 0.16 ∼ 0.33, *p* < 0.0001), 0.31 D (95% CI, 0.18 ∼ 0.44, *p* < 0.0001) and in AL from MFLs were −0.08 mm (95% CI, −0.09 ∼−0.07, *p* < 0.00001), −0.10 mm (95% CI, −0.12 ∼−0.09, *p* < 0.00001), −0.14 mm (95% CI, −0.17 ∼−0.11, *p* < 0.00001), and −0.18 mm (95% CI, −0.22 ∼−0.14, *p* < 0.00001) slower comparing with SVLs at follow up of 6, 12, 18, and 24 months, respectively.

**Conclusion:**

The treatment effects of MFLs to slow down the myopic progress are positive in both 6-months and annual-visit trials and which could be sustained till 36 months. While a slight weaker treatment effect was observed after the first visit in 6-months visit, a slight rebound was observed at the following visit points. Furthermore, the treatment effects in annual visit are more profound than 6-months visit at almost all stages especially in SER. Our analysis encourages the MFLs users to maintain a long-term treatment with annual visit.

## Introduction

High myopia (spherical equivalent, >−6.00 D) leads to the irreversible retinal atrophy and will cause a lot of complications such as macular degeneration, retinal detachment, glaucoma, and premature cataracts (1–3). The risk of suffering from a retinal detachment is 20 times more in a person with high myopia than an emmetropic one (4). Recently, the high prevalence of myopia worldwide among children and teenagers is a serious threat of public health especially in Asia (5–7). In China, the myopia incidence rate in primary school is approximately 40%, in middle and high school students is even higher and could be even severer in near future (8–10). The increasing number of myopias in teenagers whose myopia progress even faster attracts more attention to develop novel strategies to slow down the progress of myopia (11).

To slow down the progress of myopia, it is the prerequisite to understand the risk factors and mechanisms of myopia. The previous studies suggested that the genetic factors play substantial roles than the environmental ones (12, 13). Thus, the children whose parents are myopia trend to be easier to suffer from myopia than those children who have only one or no myopic parent. However, there are solid evidences also clearly showed that the environmental factors could not be ignored for developing myopia especially at current age when it requires school-aged children to take longer time reading, sustainable homework and spend fewer hours for outdoor activities (14–16).

According to animal studies, it was demonstrated that hyperopic defocus in retina causes refractive development and eye axial excessive growth, which promotes myopia progression. Conversely, myopic defocus in retina could retard the eye axial growth, which slows myopia progression (17–20). On the basis of these observations, a novel lens, namely, multifocal lens (MFL) was developed with peripheral focus technology that provides a central zone containing the distance correction and periphery zone having a myopic defocus by adding an extra positive power, resulting in myopic defocus in retina to slow down the eye axial elongation. Currently, there are two main types of MFLs designs including concentric rings/bifocals (BF) and progressive power/peripheral add lens (PAL), which principally provide both near- and distance-vision spectrum (21). With BF, it has two zones of myopia correction for all gaze positions and two neighboring concentric treatment zones with plus power addition to simultaneously deliver peripheral myopic defocus (22, 23). However, PAL simultaneously produces constantly peripheral myopization defocus that increases gradually from the central optic axis toward the periphery (21, 23).

Although it is in theory that MFLs could slow down the myopia progression, the outcomes from clinic practice are controversy. Thus, the several earlier meta-analyses also could not achieve consistent conclusion due to multiple reasons. Li et al. (24) collected nine randomized controlled trials (RCTs) from 1989–2010 to compare effects of MLCs with single vision lenses (SVLs) in children. The data suggested that MLCs with powers ranging from + 1.50 to + 2.00 D were associated with a statistically significantly decrease in myopia progression in school-aged children compared with SVLs. In 2017, the other group (24) evaluated the possible difference between BFs and PALs. They found that both BFs and PALs are clinically effective to control myopia in school-aged children. However, it seems that BFs seem to have greater effect than PALs. Recently, Kaphle et al. (25) performed a meta-analysis to compare the absolute progression rates over the successive 6-months or 1-year periods to gauge how long the efficacy of the intervention lasts. They found that the treatment effect of MFLs is maximum in the first 6- and 12-months intervals and is reduced in subsequent intervals. A latest study (26) also suggested a similar tendency when comparing the relative increases rather than absolute increases in measures of myopia progression for PALs and SVLs that the relative efficacy of PALs tends to be weaker after the first 12 months. Overall, these studies indicate that MFL is effective to slow down the progress of myopia in children, but how long the treatment effects could be sustained and whether treatment interval affects the effects remain to be intensively reviewed.

In this study, we conducted a systematic meta-analysis focusing on currently available evidences from 15 high quality RCTs involving 1,840 children aged 6–18 years to assess the effects of MFLs vs. SVLs on slowing myopic progress. We extended our analyzed treatment period up to 36 months and subdivide into 6- and 12-months (annual) visit intervals. To more precisely evaluate the treatment effects, both spherical equivalent refraction (SER) and axial length (AL) are analyzed.

## Materials and Methods

### Search Strategy

A search was performed in PubMed, MEDLINE, Embase, Web of Science, and Cochrane Library (up to July 2021) using the following keywords: Myopias (MeSH Terms), Near sighted*, short sight*; eyeglasses (MeSH Terms), spectacles, single vision lenses; multifocal (MeSH Terms), bifocal, progressive addition lenses; RCT (MeSH Terms), controlled clinical trials, randomized, clinical trials, randomized, trials, randomized clinical, clinical trial. We used the Boolean operator “AND” to combine all search sets as the final step. The articles performed in “humans” and published in “English” language were used as filters.

### Studies Selection

Relevant clinical trials were selected according to the following criteria: (1) Study type: RCTs, (2) Participants: 6–18-year-old school-aged children with myopia, (3) Interventions: MFL or bifocal lens or progressive additional lens as the experimental group, and single vision soft contact lenses or spectacles as the control group, and (4) Primary outcomes: the change in refractive errors (cycloplegic SER), that is, myopia progression, with 95% confidence interval (CI) or standard deviation and the change in axial elongation (AL) compared with the baseline at different visits. The exclusion criteria were as follows: (1) Duplicates; (2) Studies with missing information; (3) Studies that were published earlier than 2000; (4) Corresponding authors could not be contacted for missing information; (5) Articles that were not published in English; (6) Myopia progression measured in participants who wore contact lenses or orthokeratology or were using eye drops; and (7) Review articles, case studies, and cross-sectional studies.

### Data Extraction and Quality Assessment

The data were independently extracted including the following information: Authors, publication year, country or area, type of multifocal lenses, age and sex of the study population, sample size, proportion lost to follow-up, length of follow-up, myopia progression with standard deviation at 6- or 12-months intervals, and information on methodology. For the studies with missing information, an email was sent to the corresponding authors who supplied additional data, if needed, used GetData Graph Digitizer 2.24^1^ to read data of different follow-up periods, which were only illustrated in figures. For studies that provided baseline and final SER and standard deviation, but not the standard deviation of the change, an equation suggested by Cochrane collaboration (27) was used to calculate the standard deviation of the change.

Quality assessment of the included studies was performed by the Newcastle–Ottawa Quality Assessment Scale items. This includes 16 items with the following three domains: Selection (representativeness), comparability (because of design or analysis), and outcomes (assessment and follow-up). One study can be awarded a maximum of one star for each numbered item within the selection and outcome categories. A maximum of two stars can be given for comparability. Any discrepancy between the two reviewers about the above issues was resolved by discussion or a third reviewer.

### Statistical Analysis

All statistical analyses were conducted by Review Manager, version 5.3.^2^ The differences in refraction and AL between the two groups were assessed as mean differences (experimental group minus control group, Cochrane Handbook 5.1.0, 9.2.3.1) and 95% CI. The random effects analysis method was used for meta-analysis when there was significant heterogeneity between studies. Statistical heterogeneity in articles was assessed with the *I*^2^ statistic, with *I*^2^ > 50%, and p < 0.1 considered to indicate high heterogeneity. The sensitivity analyses were performed by sequentially removing the individual studies to determine whether each resulted in a substantial change in the magnitude or direction of the pooled estimates and heterogeneity. When the excluded study substantially changed the mean difference in SER and *I*^2^ value, it is reported in the results. Statistical significance was declared as *p* < 0.05.

## Results

### Characteristics of Studies Included in the Meta-Analysis

**FIGURE 1:**
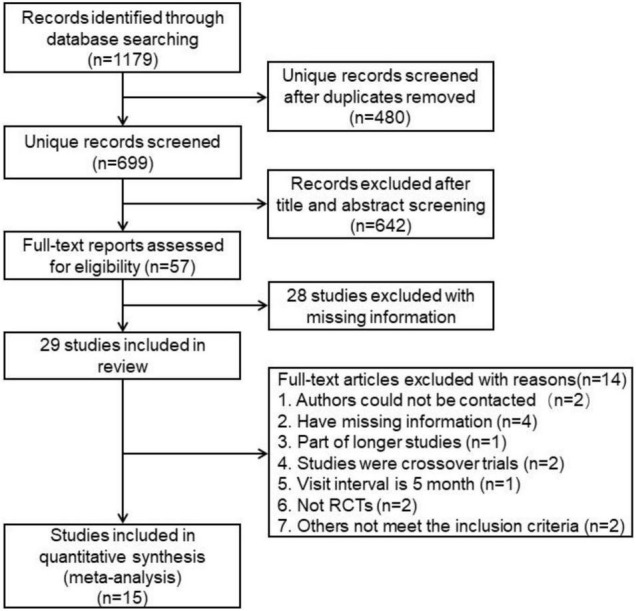
A flowchart of study selection.

A flowchart of study selection is presented in Figure 1. Totally 1,179 studies were identified from the search using PubMed, Cochrane, EMBASE, MEDLINE and Web of Science. After removing the duplicates, there were 699 studies remained. By reviewing the title and abstract, 642 studies were excluded and 57 studies were remained. After a full-text review, 29 studies were included. Among these 29 remaining trails, 13 studies were excluded for the following reasons: Five studies had missing information (28–32), authors of two studies could not be contacted for missing information (33, 34), one study was a part of a longer study (35). Two studies was not randomized control trial (36, 37), one trail was recorded with 5-months interval (38), one trial was crossover trial (39), one trial recorded only one outcome (40), and in one study, the control group switched to MFLs at the second year (41).

The characters of 15 included studies are showed in Tables 1, 2. It was conducted on 1,840 children aged between 6 and 18 years. The time length of the follow-ups varied from 12–36 months as follows: Four studies were of 12-months duration (42–45), seven studies were of 24-months duration (22, 46–51), and four studies were of 36-months duration (33, 52–54). Four studies were conducted in the United States America (48, 51, 52, 54). Two studies were multi-country studies as follows: The trial from Chamberlain et al. was conducted in (Portugal, United Kingdom, Singapore, Canada) and the study of Hasebe et al. was carried in China, Japan, and South Korea (50, 53). Two studies were carried out in Spain (22, 42), five studies were carried out in China (44–47, 49) and the rest of the studies were conducted in the following countries: Australia (43) and Canada (55). Eight studies used bifocals (BFs), one used novel lens designs that corrected hyperopic defocus partly or fully in the periphery, and the other six used PALs as the intervention. For the relative peripheral positive powers, + 3.50 D was used in one study (46), + 2.5 D was used in four studies (42, 47, 49, 52), + 2.00 D was used in six studies (22, 36, 44, 45, 51, 54), + 1.9 D was used in one study (45), + 1.50 D were used in three studies (47, 52, 55), and + 1.0 D was used in one study (45).

**TABLE 1:** Characteristics of included annual-visit studies in meta-analysis.

Study	Location	Intervention/Control	Age of participants (years)	No. of participants	Female (%) MSLs SVLs	Drop-out N (%) MSFs SVLs	Follow-up, months	Baseline SER
Walline et al. (52)	United States	BF a: H:add + 2.5 Day SVL	(7–11 years) 10.2 ± 1.2 10.5 ± 0.96	196 (98 + 98)	64 (65.3) 64 (65.3)	12 (4.1)	36	−0.75 ∼−5.0 Day −2.28 ± 0.9 Day −2.46 ± 0.97
Chamberlain et al. (53)	Portugal + United Kingdom + Singapore + Canada	Misight, BF add + 2.0 Day SVL	(8–12 years) 10.1 ± 1.3 10.1 ± 1.4	144 (70 + 74)	32 (46) 37 (50)	12 (24.2) 18 (24.3)	36	−0.75 ∼−4.0 Day −2.02 ± 0.77 −2.19 ± 0.81
Ruiz-Pomeda et al. (22)	Spain	Misight, BF add + 2.0 Day SVL	(8–12 years) 11.01 ± 1.23 10.12 ± 1.38	79 (46 + 33)	NA	5 (10.9) 0 (0)	24	−0.75 ∼−4.0 Day −2.16 ± 0.94 −1.75 ± 0.94
Cheng et al. (55)	Canada	BF a: add + 1.5 Day, b: Prismatic, add+1.5 Day + 3^–▲^ SVL	(8–13 years) 10.1 ± 0.2 10.4 ± 0.3 10.3 ± 0.3	150 (50 + 50 + 50)	24 (48) 25 (50) 24 (48)	2 (4) 4 (8) 0 (0)	36	>1.0 Day −3.08 ± 0.1 Day
Walline et al. (51)	United States	BF, add + 2.0 Day SVL	(8–11 years) 10.8 ± 0.7 10.8 ± 1.0	80 (40 + 40)	18 (56.3) 18 (56.3)	13 (26) 13 (26)	24	−1.0 ∼−6.0 Day −2.35 ± 1.05 −2.24 ± 1.02
COMET (54)	United States	PAL add + 2.0 Day SVL	(8–12 years) 10.2 ± 1.1 10.0 ± 1.1	118 (59 + 59)	33 (63.5) 27 (47)	7 (11.86) 1 (1.69)	36	−0.75 ∼−2.5 Day −1.45 ± 0.47 −1.5 ± 0.45

*BF, bifocal; PAL, progressive addition lens; SVLs, single-vision lenses; NA, not available.*

**TABLE 2:** Characteristics of included 6-months visit studies in meta-analysis.

Study	Location	Intervention/Control	Age of participants (years)	No. of participants	Female (%) MSLs, SVLs	Drop-out N (%) MSFs SVLs	Follow-up, months	baseline SER
Lam et al. (46)	China	DIMS, BF, Relative peripheral power + 3.5 Day SVL	8–13 years 10.2 ± 1.47 10 ± 1.45	183 (93 + 90)	41.80 45.70	14 (15) 9 (10)	24	−1.0 ∼−5.0 Day −2.97 ± 0.97 −2.76 ± 0.96
Garcia-Del Valle et al. (42)	Spain	PALs, add + 2.5 Day SVL	(7–15 years) 12.2 ± 2.22 11.9 ± 2.13	70 (36 + 34)	19 (59.4) 18 (69.2)	4 (11.1) 8 (23.5)	12	−0.5 ∼−8.75 Day −2.8 ± 1.79 −3.31 ± 1.76
Sankaridurg et al. (47)	China	Relative peripheral power a: I add + 2.5 Day, b: II add + 1.5 Day, SVL	(8–13 years) 10.4 ± 1.3 10.4 ± 1.3 10.5 ± 1.3	306 (103 + 101 + 102)	49 (47.6) 52 (51.5) 43 (42.2)	56 (54.4) 56 (55.4) 52 (50.1)	24	−0.75 ∼−3.5 Day −2.38 ± 0.82 −2.39 ± 0.79 −2.29 ± 0.75
Cheng et al. (48)	United States	+ SA SVL	(8–11 years) 9.7 ± 1.11 9.7 ± 1.05	127 (64 + 63)	24 (45.3) 27 (45.8)	11 (17.2) 4 (6.8)	24	−0.75 ∼−4.0 Day −2.52 ± 1.094 −2.44 ± 0.911
Aller (43)	Australia	BF SVL	(8–18 years) 13.0 ± 2.5 13.5 ± 2.2	86 (43+ 43)	27 (62.8) 27 (62.8)	4 (9.30) 3 (6.98)	12	−0.5 ∼−6.0 Day −2.57 ± 1.34 −2.81 ± 1.46
Lam et al. (49)	China	DISC, BF add + 2.5 Day SVL	(8–13 years) 11.06 ± 1.55 10.87 ± 1.67	221 (111 + 110)	44 (67.7) 39 (61.9)	46 (41.4) 47 (42.7)	24	−1.0 ∼−5.0 Day −2.9 ± 1.05 −2.80 ± 1.03
Hasebe et al. (50)	China Japan	PAL, a: add + 1.0 Day b: add + 1.5 Day SVL	(6–12 years) 10.6 ± 1.50 10.0 ± 1.50 10.2 ± 1.20	197 (67 + 67 + 63)	30 (45) 20 (32) 24 (36)	9 (13) 12 (19) 710)	24	−1.0 ∼−4.5 Day −2.52 ± 1.01 −2.80 ± 1.02 −2.55 ± 0.96
Sankaridurg et al. (44)	China	PAL, add + 2.0 Day SVL	(7–14 years) 11.6 ± 1.5 10.8 ± 1.9	100 (60 + 40)	23 (51) 17 (43)	17 (28.3)1 (2.5)	12	−0.75 ∼−3.5 Day −2.9 ± 1.05 −2.8 ± 1.03
Sankaridurg et al. (45)	China	Relative peripheral power a:Type I add + 1.0 Day b:Type II add + 2.0 Day c:Type III add + 1.9 Day SVL	(6–16 years) 10.7 ± 2.4 11.1 ± 2.2 11.4 ± 2.3 10.0 ± 1.1	210 (50 + 60 + 50 + 50)	27 (54) 26 (43) 25 (50) 22 (44)	2 (4) 2 (3) 4 (8) 1 (2)	12	−0.75 ∼−3.5 Day −1.82 ± 0.62 −1.81 ± 0.67 −1.82 ± 0.66 −1.87 ± 0.68

*BF, bifocal; PAL, progressive addition lens; SVLs, single-vision lenses.*

### Trial Quality

Quality assessment of the included studies was performed by the Newcastle–Ottawa Quality Assessment Scale items (Table 3). This includes 15 items with following three domains: Selection (representativeness), comparability (because of design or analysis), and outcomes (assessment and follow-up). One study can be awarded a maximum of one star for each numbered item within the selection and outcome categories. A maximum of two stars can be given for comparability. The quality of the included RCTs was generally high, the scores from all trials are ≥6.

**TABLE 3:** Quality assessment of cohort studies included in the meta-analysis using Newcastle–Ottawa quality assessment scale.

	Selection					Outcome			
	**Exposed cohort representative**	**Non-exposed cohort selection**	**Exposure ascertainment**	**Outcome not present at star**	**Comparability of cohorts**	**Assessment**	**Follow-up length**	**Follow-up adequacy**	**NOS score**
**Study**									
Lam et al. (46)	★	★	★	★	★★	★	★	★	9
Garcia-Del Valle et al. (42)	★	★	★	★	★★	★	★	★	9
Sankaridurg et al. (47)	★	★	★	★	★★	★	★	✩	8.5
Cheng et al. (48)	★	★	★	★	★★	★	★	★	9
Aller (43)	★	★	★	★	★★	★	✩	★	8.5
Lam et al. (49)	★	★	★	★	★★	★	★	✩	8.5
Hasebe et al. (50)	★	★	★	★	★★	★	★	✩	8.5
Sankaridurg et al. (44)	★	★	✩	★	★	✩	★	★	7
Sankaridurg et al. (45)	★	★	★	★	★★	✩	★	★	8.5
Walline et al. (52)	★	✩	★	★	★★	★	★	★	8.5
Chamberlain et al. (53)	★	★	★	★	★★	★	★	✩	8.5
Ruiz-Pomeda et al. (22)	★	✩	★	★	★	★	★	★	7.5
Cheng et al. (55)	★	★	✩	★	★	✩	★	★	7
Walline et al. (51)	★	✩	✩	★	★	✩	★	✩	6
COMET (54)	★	★	★	★	★★	✩	★	★	8.5
									

### The Risk of Bias of the Including Trials

**FIGURE 2:**
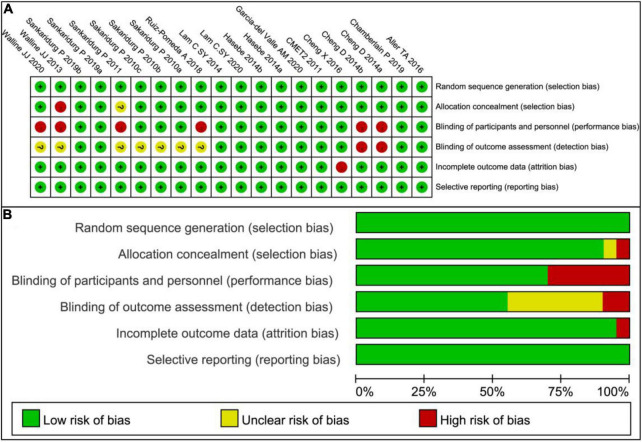
Risk of bias graph. **(A)** Risk of bias summary: Review authors’ judgments about each risk of bias item for each included study. **(B)** Risk of bias graph: Review authors’ judgments about each risk of bias item presented as percentages across all included studies.

The risk of bias (Figures 2) of the included studies was assessed by Revman 5.3 according to the following points: (1) Random sequence generation (selection bias); (2) Allocation concealment (selection bias); (3) Blinding of participants and personnel (performance bias); (4) Blinding of outcome assessment (detection bias); (5) Incomplete outcome data (attrition bias); (6) Selective reporting (reporting bias); and (7) Other bias. The masking was not adequate in four studies (22, 44, 51, 55) and no allocation concealment was present in one paper (51). In general, the risks of these 15 trials were low (Figure 2).

### Treatment Effects of Multifocal Lens Assessed for Spherical Equivalent Refraction and Axial Length

The mean differences of SER and AL between MFLs and SVLs were calculated. Since the part of the subgroups shows moderate heterogeneity (*I*^2^ > 50% and *p* < 0.05) across the studies, we analyzed the data with a random-effect model as the previous studies did.

**FIGURE 3:**
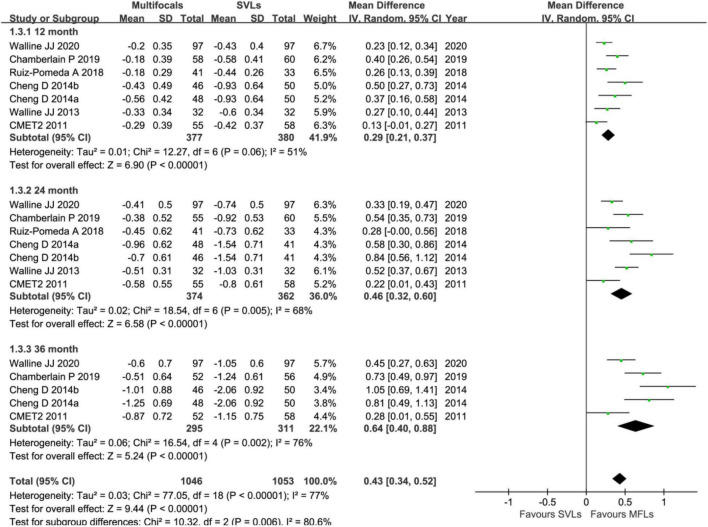
Forest plot about the change of SER in annual interval visit trials.

**FIGURE 4:**
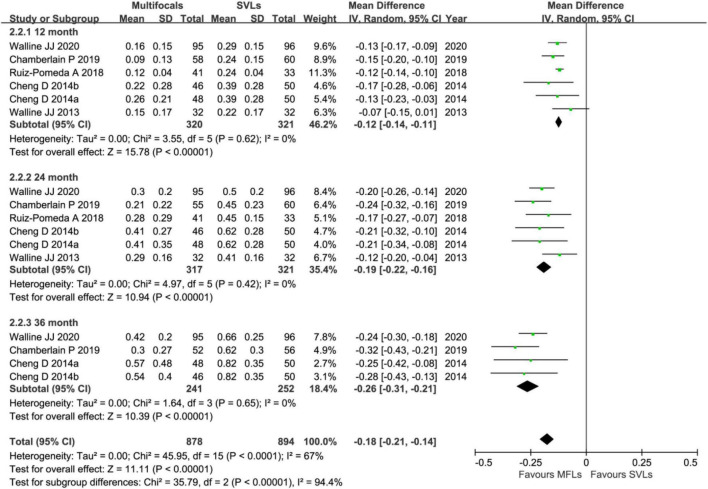
Forest plot about the change of AL in annual interval visit trials.

Annual (12-months) visit group includes six trials and among which four trials finished 3 years’ follow up (Figure 3). By analyzing SER in this group, the weighted mean differences (WMDs) of myopic progresses with MFLs are 0.29 D (95% CI, 0.21 ∼ 0.37, *p* < 0.00001) slower than SVLs at first year, 0.46 D (95% CI, 0.32 ∼ 0.60, *p* < 0.00001) and 0.64 D (95% CI, 0.40 ∼ 0.88, *p* < 0.00001) at the second and third year, respectively. For the AL (Figure 4), the myopic progresses with MFLs are −0.12 mm (95% CI, −0.14 ∼−0.11, *p* < 0.00001), −0.19 mm (95% CI, −0.22 ∼−0.16, *p* < 0.00001), and −0.26 mm (95% CI, −0.31 ∼−0.21, *p* < 0.00001) less compared to SVLs at the first, second, and third year. With annual visit subgroup, we could conclude that the inhibition of the myopic progress of MFLs in both SER and AL are significant and sustained at least for 3 years, which is different with the previous analysis (25, 26). Heterogeneity of this subgroup analysis are moderate in SER (12 months: *p* = 0.06, *I*^2^ = 51%; 24 months: *p* = 0.005, *I*^2^ = 68%; 36 months: *p* < 0.002, *I*^2^ = 76%) and none in AL (12 months: *p* = 0.62, *I*^2^ = 0%; 24 months: *p* = 0.42, *I*^2^ = 0%; 36 months: *p* = 0.65, *I*^2^ = 0%). When the prismatic bifocal intervention group of the study by Cheng et al. (55) was excluded from the SER analysis, the heterogeneity (*I*^2^) reduced to 39% (*p* = 0.14), 51% (*p* = 0.07), and 69% (*p* = 0.02) at 12-, 24-, and 36 months, respectively. The mean differences reduced to 0.27 D (95% CI, 0.19 ∼ 0.34, *p* < 0.00001), 0.41 D (95% CI, 0.30 ∼ 0.53, *p* < 0.00001), and 0.56 D (95% CI, 0.34 ∼ 0.78, *p* < 0.00001) at 12-, 24-, and 36 months, respectively.

**FIGURE 5:**
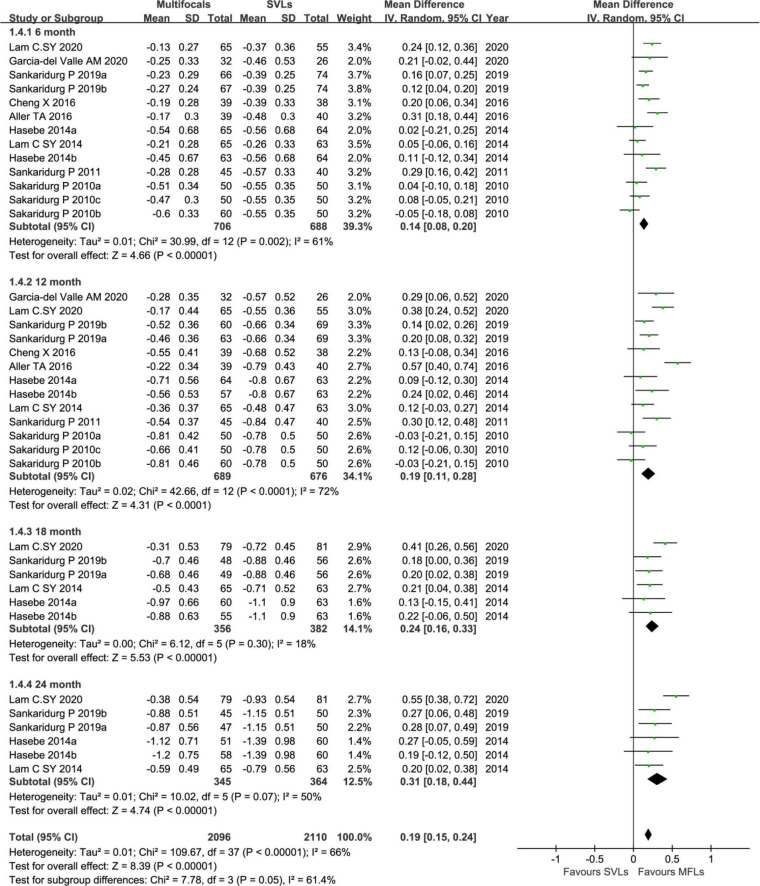
Forest plot about the change of SER in 6-months interval visit trials.

**FIGURE 6:**
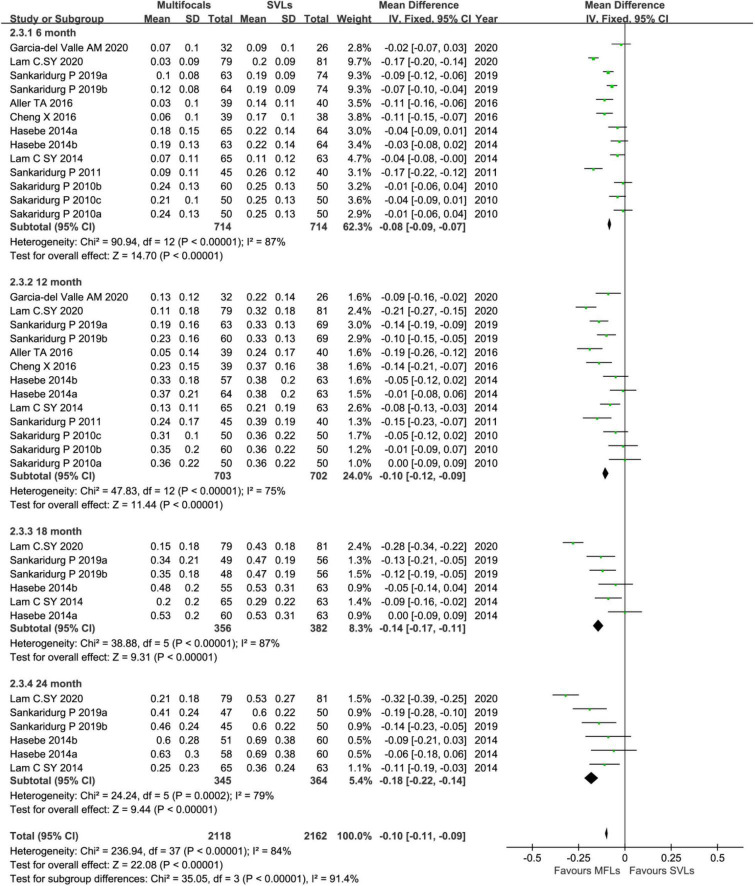
Forest plot about the change of AL in 6-months interval visit trials.

On analyzing SER in 6-months interval visit group (Figure 5), the inhibition levels of the myopic progress with MFLs are 0.14 D (95% CI, 0.08 ∼ 0.20, *p* < 0.0001), 0.19 D (95% CI, 0.11 ∼ 0.28, *p* < 0.0001), 0.24 D (95% CI, 0.16 ∼ 0.33, *p* < 0.0001), and 0.31 D (95% CI, 0.18 ∼ 0.44, *p* < 0.0001) at 6, 12, 18, and 24, respectively. For AL reduction levels with MFLs in this group (Figure 6) are −0.08 mm (95% CI −0.09 ∼−0.07, *p* < 0.00001) at 6 months, −0.10 mm (95% CI −0.12 ∼−0.09, *p* < 0.00001) at 12 months, −0.14 mm (95% CI −0.17 ∼−0.11, *p* < 0.00001) at 18 months and −0.18 mm (95% CI −0.22 ∼ -0.14, *p* < 0.00001) at 24 months. We could show that a slightly decreased treatment effect was observed after first visit in both SER and AL, which is consistent with the previous meta-analysis (25, 26). However, we also observed an obvious rebound in AL at the following data points. A small or moderate heterogeneity in SER (6 months: *p* = 0.002, *I*^2^ = 61%; 12 months: *p* < 0.0001, *I*^2^ = 72%; 18 months: *p* = 0.3, *I*^2^ = 18%, 24 months: *p* = 0.07, *I*^2^ = 50%) and a moderate or high heterogeneity in AL analysis (6 months: *p* < 0.00001, *I*^2^ = 87%; 12 months: *p* < 0.00001, *I*^2^ = 75%; 18 months: *p* < 0.00001, *I*^2^ = 87%; 24 months: *p* = 0.0002, *I*^2^ = 79%) of this subgroup is detected. When excluding the study with defocus incorporated multiple segments (DIMS) spectacle lenses in this subgroup from Lam et al. (46), the heterogeneity reduced to a tolerable level or zero in both SER (6 months: *p* = 0.004, *I*^2^ = 60%; 12 months: *p* < 0.0002, *I*^2^ = 69%; 18 months: *p* = 0.99, *I*^2^ = 0%, 24 months: *p* = 0.99, *I*^2^ = 0%) and AL (6 months: *p* < 0.00001, *I*^2^ = 76%; 12 months: *p* = 0.0007, *I*^2^ = 66%; 18 months: *p* = 0.19, *I*^2^ = 34%; 24 months: *p* = 0.47, *I*^2^ = 0%). The mean differences of SER (6 months: 0.13 mm, 95% CI 0.07 ∼ 0.19, *p* < 0.00001; 12 months: 0.18 mm, 95% CI 0.09 ∼ 0.27, *p* = 0.00001; 18 months: 0.19 mm, 95% CI 0.10 ∼ 0.28, *p* < 0.0001; 24 months: 0.24 mm, 95% CI 0.14 ∼−0.34, *p* < 0.00001) and AL (6 months: −0.06 mm, 95% CI −0.09 ∼−0.04, *p* < 0.00001; 12 months: −0.09 mm, 95% CI −0.12 ∼−0.05, *p* < 0.00001; 18 months: −0.08 mm, 95% CI −0.13 ∼−0.04, *p* = 0.0002 and 24 months: −0.13 mm, 95% CI −0.17 ∼−0.08, *p* < 0.00001) are also reduced a little bit accordingly but without altering the conclusions.

In short summary, our systematic analysis clearly showed that the treatment effects of MFLs to slow down the myopic progress are positive in both 6-months and annual-visit trials and which could be sustained to 36 months. While a slight weaker treatment effect was observed after the first visit in 6-months visit, a slight rebound was observed at following visit points. Furthermore, the treatment effects in annual visit are more profound than 6-months visit at almost all stages especially in SER although this trend did not reach the statistic difference likely due to the enrolled number of annual studies is not enough. Furthermore, this observation is not altered when excluding the related studies to reduce the heterogeneity, indicating the conclusion is in principle acceptable. Thus, our analysis encourages the MFLs users to maintain a long-term treatment with annual visit.

## Discussion

Currently, the most prevalent treatments to control the myopia progress involves in pharmacological agents such as atropine ophthalmologic drops, orthokeratology (OK), and MFL. Atropine ophthalmologic drops could reduce accommodation and increase pupillary diameter, resulting in well-controlled myopia progress (56). However, because of its side-effects such as photophobia, poor near visual acuity, increased pupillary diameter, and headache, many parents terminate the treatment with atropine for their children. In addition, the mechanism why atropine could control myopic progress and whether long-term application of antimuscarinic agents on ocular tissue is harmful or not remain to be determined (38). Orthokeratology is suitable for the low-to-moderate myopia, the children can wear it overnight to remodel the corneal epithelial into a flatter and less powerful refractive surface, achieving a transient emmetropia (57, 58). According to the clinical trials, the axial elongation with OK group could be significantly inhibited and the peripheral refractive status of the cornea could be less hyperopic defocus when comparing with single vision group (59). However, the children who take OK treatments are easy to suffer from microbial keratitis, contact lens irritation, dry eye, and corneal epithelial iron deposition (60, 61).

**FIGURE 7:**
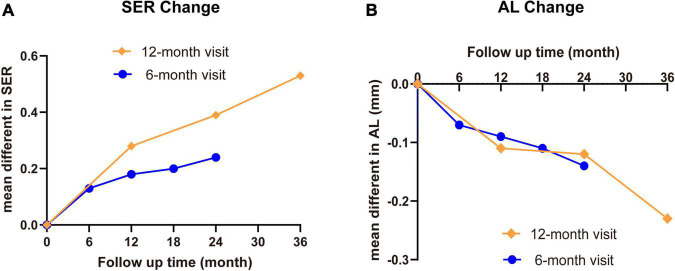
The summary of SER and AL change with MFL treatment in school-aged children. The mean differences of SER **(A)** and AL **(B)** between MFLs (intervention) and SVLs (control) with different visit intervals were summarized.

In this study, we summarized currently available evidences of controlling myopia progression in school-aged children aged 6–18 years from 15 RCTs to dissect the potential roles of MFLs treatment. The effects of MFLs to slow down the myopic progress could be sustained to 36 months but with slightly decreased effects from the second visit. While the treatment effects in both 6-months and annual-visit trials are positive, the annual visit is more profound comparing to 6-months visit group (Figure 7) although no statistic difference was detected which is likely due to the variability and small samples.

It is still unknown why a decreased effect happened after first visit in both 6-months visit and annual visit trials not only in our analysis but also in the previous reports (25, 26). Our analysis showed that in annual visit, differences of mean SER change is 0.29 D for the first year, 0.17 D for the 12–24 months and 0.18 D for the 24–36 months; of mean AL change is −0.12 mm for the first year, −0.07 mm for the 12–24 months and −0.17 mm for the 24–36 months. In 6-months visit, the difference of mean SER change is 0.14 D for the first visit and around 0.05–0.07 D for the following visit intervals; of mean AL change is −0.07 mm for the first visit and −0.04 to −0.02 mm for the following visits. Kaphle et al. proposed accommodation adaptation may play a role to explain this phenomenon (25). It was reported that the lag of accommodation gradually increases while a person wearing MFLs (62), the same addition power may not be as effective as it did earlier. Therefore, to maintain the effective treatment for MFLs as they were in the beginning of the trial, the power of the addition should be increased gradually. In addition, they also supposed that the age is a critical factor since myopia usually stabilizes when a child reaches a certain age which means the rate of myopia progression decreases over time in the SVLs control group, and hence the treatment effect of the myopia intervention reduces as it is determined *via* comparison with the progression of the SVLs group. This argument is however not very reliable since the average age of children involved in these clinic trails is around 9.5–12 years old at least in current study (Tables 1, 2), which is far from the stage for the myopia stabilization although a slower and more stable rates of change of myopia after onset was observed (63, 64).

In addition, we clearly present an obvious difference in distinct treatment intervals. Comparing to 6-months trails, the annual visit with MFLs show more profound effects [annual vs. 6-months visit: SER: 0.29 D (95% CI 0.21 ∼ 0.37) vs. 0.19 D (95% CI 0.11 ∼ 0.28) at the first year, 0.46 D (95% CI 0.32 ∼ 0.60) vs. 0.31 D (95% CI 0.18 ∼ 0.44) at the second year; AL: −0.12 (95% CI −0.14 ∼−0.11) vs. −0.07 (95% CI −0.10 ∼−0.04) at the first year, −0.19 (95% CI −0.22 ∼ -0.16) vs. −0.10 (95% CI −0.13 ∼−0.06) at the second year]. One possibility could be that frequent lens power adjustment (less than 1 year) affects the accommodation adaptation response which eventually blocks the treatment effects. Thus, the detailed mechanisms on why frequent lens power adjustment is not conducive to myopia control in this case should be further explored.

There are several limitations in our study. First, only the RCTs were included in the analysis. We excluded at least one clinic research from Paune et al. (36) which is a prospective, longitudinal, non-randomized study (36). In addition, a few studies, for example the trials from Fujikado et al. (39) and Berntsen et al. (41) met the inclusion criteria, but were not included because of unavailability of data at the required time points.

In addition, much fewer (only 4 in 15 RCTs) studies finished 3-year observation. One trial from Lam et al. (46) was a part of the data from Lam (35) by year of 2021. However, the data at 36 months from the trail of Lam et al. (35) was discarded because it lacks of control data since the control group of this trial was switched from SVLs to MFLs at this time point. Besides, we could not exclude the possibility that the third-year interval was terminated due to either good or no obvious treatment effects at the first and/or especially the second year, which could affect the treatment effects in the meta-analysis. In this analysis, we could reach the data of 3-year treatments with annual visit but not 6-months visit. Therefore, the difference between annual and 6-months visit for a long-term treatment effect is not determined.

Third, our analysis may also have publication bias since the studies we selected were peer-reviewed. Currently, the studies with positive treatment effects are much easy to be published comparing to the studies with negative or no obvious treatment effects. Besides, some clinic trails may be terminated because no significant treatment effects are observed at early stage. This likely would overestimate the treatment effect of intervention.

Lastly, heterogeneity is a common problem for meta-analysis and it is difficult to deal with especially when the enrolled studies is selected in certain cases. In current study, we noticed a high heterogeneity which promotes us to do subgroup analysis. By analyzing the potential factors such as the county/region, added power and visit intervals (6-months and annual-visit), we found that subgroup with the visit intervals shows smaller and tolerable heterogeneity. Actually, this factor is exactly the one we would like to analyze because it seems distinct visit intervals did affect the treatment effects from literatures and in clinic practice. However, the part of data still shows moderate heterogeneity after subgroup. Therefore, a random-effect model for analysis was selected as other previous studies conducted. In addition, it is also possible to eliminate/reduce the heterogeneity by excluding certain studies. Thus, we found that by excluding the studies from Lam et al. (46) and Cheng et al. (55) could significantly reduce the heterogeneity. However, excluding specific study is not applicable when no decent reasons arising. Nevertheless, we found that the mean differences when including or excluding these two studies are only tiny changed, which did not alter any of the interpretations. Furthermore, a meta-regression could be also performed to find the potential factors that arise the heterogeneity. However, the meta-regression would not eventually dissolve the problem since we have failed to reduce the heterogeneity when subgroup with country/region and added power and we are mainly focusing on the treatment effects from distinct visit intervals in current study.

In conclusion, our analysis showed that the treatment effect of MFLs is positive in either 6-months or annual visit although annual visit shows more profound effects. In addition, it also highlights that while a slightly decreased effect was observed from the second visit in both 6-months and annual visit, a longer treatment likely acquires a better effect. Thus, the data encourages the MFLs users to maintain the long-term treatment (at least for 36 months) with annual visit. Due to the presence of heterogeneity in this analysis, a standardized large scale multi-center clinical trial should be conducted to provide an explicit direction.

## Data Availability Statement

The original contributions presented in this study are included in the article/supplementary material, further inquiries can be directed to the corresponding authors.

## Author Contributions

MC and YW designed and supervised the project. MC performed literature review, data analysis, figure preparation, and prepared the manuscript with contributions from all authors. LX and CH prepared the tables. HL, FC, and HW reviewed the literatures and datasets. All authors contributed to the article and approved the submitted version.

## Conflict of Interest

The authors declare that the research was conducted in the absence of any commercial or financial relationships that could be construed as a potential conflict of interest.

## Publisher’s Note

All claims expressed in this article are solely those of the authors and do not necessarily represent those of their affiliated organizations, or those of the publisher, the editors and the reviewers. Any product that may be evaluated in this article, or claim that may be made by its manufacturer, is not guaranteed or endorsed by the publisher.

## Footnotes

1
http://getdata-graph-digitizer.com

2
http://tech.cochrane.org/revman
